# Non vitamin K anticoagulation drugs will win the race

**DOI:** 10.1007/s12471-016-0895-6

**Published:** 2016-09-06

**Authors:** I. C. Van Gelder, R. R. De With, M. Rienstra

**Affiliations:** Department of Cardiology, University of Groningen University Medical Center Groningen, Groningen, The Netherlands

Atrial fibrillation (AF) is associated with major adverse cardiovascular and cerebral events, including stroke. In order to prevent stroke, oral anticoagulation (OAC) therapy is prescribed guided by risk-stratification scores, nowadays the CHA_2_DS_2_-VASc score [1]. For more than 50 years vitamin K antagonists (VKA) have had a central role in stroke prevention in patients with AF. Most important limitations of VKAs, however, include the need for frequent monitoring of the international normalised ratio (INR) and dosing changes. Despite repeated assessment of the INR and dosing changes, it remains difficult to achieve therapeutic ranges in a substantial number of patients due to drug and food interactions**,** poor adherence or other, yet unknown, factors [2]. Non vitamin K oral anticoagulation drugs (NOAC) are now available as an alternative to VKA [3–6]. Many AF patients who qualify for OAC treatment are candidates for these drugs. However, despite fast adoption of these new drugs in other countries, the prescription rate in the Netherlands is still lower than expected.

In this issue of the Netherlands Heart Journal, Ten Cate et al. describe the use of NOACs in the Netherlands in order to provide information on the prescription behaviour of OACs in patients with AF [7]. Data are shown from the Global Anticoagulant Registry in the FIELD-Atrial Fibrillation (GARFIELD-AF), a worldwide multicentre registry on anticoagulants use in patients with AF. A total of 41,677 patients with new-onset AF (<6 weeks) with at least one additional risk factor for stroke were included in 35 countries; 929 of them were enrolled in 16 Dutch centres. Patients were included in three different cohorts during three successive years of inclusion. One finding of interest of this registry is between 4.4 % and 7.3 % did not use any kind of antithrombotic drugs and a comparable number of patients only used antiplatelet therapy. Further, they observed a gradual but small increase in the NOAC prescription rate in the successive cohorts. In the first cohort (December 2009–October 2011) none of the patients used a NOAC because none of these drugs had been approved in the Netherlands at that time. In the third and last cohort (inclusion June 2013–June 2014), however, 14.5 % of the patients used a NOAC, which is higher than the NOAC prescription rate in the second cohort (2.7 %, inclusion October 2011 and June 2013). However, this number is still significantly lower compared with other parts of the world (40 % in the third cohort). The authors’ explanation for the current low NOAC prescription rate in the Netherlands is the presence and structure of well-organised specialised clinics for VKA monitoring in our country. These clinics facilitate INR monitoring and dosing changes. Other countries rarely have a structure in which these tasks are facilitated. Further, three of these specialised clinics enrolled patients for the GARFIELD, which may also have contributed to the relatively high proportion of patients using VKA and the slow uptake of NOACs in this registry.

The authors are congratulated on sharing these data with us. This gives an indication of the prescription of OAC in the Netherlands until June 2014. It highlights two hurdles we encounter when prescribing OACs in patients with AF. First, a dilemma in starting OAC is always to balance efficacy of stroke prevention and safety in terms of side effects – especially major bleeding. Prescription of OAC in daily practice is not always consistent with current guidelines, as has been demonstrated by data from the present study and which approves data from the Euro Heart Survey and the EURObservational Research Programme [8, 9]. Also other data confirm the inadequate prescription of OAC in patients at risk for stroke. An analysis of the first 10,000 patients from the GLObal RegIstry on long-term oral Anti-thrombotic treatment in patients with Atrial Fibrillation (GLORIA-AF) showed that 17.6 % of all patients with a CHA_2_DS_2_-VASc score ≥2 did not use adequate antithrombotic therapy [10]. These numbers, however, greatly differed per region, from 8.1 % in Europe up to 37.6 % in Asia. The other way around, remarkably the same registry showed that 38.7 % of the patients with a CHA_2_DS_2_-VASc score of 0 did receive antithrombotic therapy [11].

A second dilemma highlighted by the authors is whether or not to start with a NOAC instead of the prescribing the well-known VKAs. Since the first NOAC approval of the direct thrombin inhibitor dabigatran for the Dutch market in 2011, three other NOACs, direct factor Xa inhibitors (rivaroxaban, apixaban and edoxaban), have been introduced. Large phase III clinical trials have all shown NOACs to be non-inferior, sometimes even superior, in terms of prevention of stroke and systemic embolic events in patients with AF and comparable rates of major bleeding [3–6]. In addition, all NOAC trials showed a 50 % reduction in intracranial haemorrhage rate compared with VKAs. Meta-analysis of these trials confirm the non-inferiority of NOACs and sometimes even suggest a superior effect in stroke prevention and systemic embolism [12]. Thus, the available data now show that NOACs are safe and effective drugs. In addition, their pharmacological properties allow NOACs to be given at fixed doses without the need for laboratory monitoring except for renal function and few drug interactions exist. Despite these data, a NOAC prescription rate of 7.4 % in the Netherlands was observed in 2015, which is second-lowest rate in Europe. Countries such as Germany, Greece and Switzerland have rates of over 40 %.

How can the low prescription rate of NOACs in our, often open-minded country, be explained? It may relate to the previously mentioned specialised clinics for VKA management. In other countries, prescribing VKA increases the workload for physicians, in contrast to the Netherlands. Here, institution of NOACs causes an increased and new, yet unfamiliar workload. Another possible explanation is the inability to monitor the anticoagulation effect, which makes the prescribing physician nervous and reluctant, especially in patients with an expected poor drug adherence. Lack of an antidote may be another reason not to prescribe NOACs. Although its relevance can be discussed, this issue is now partly resolved since the first NOAC antidote for dabigatran has been approved [13]. However, perhaps the biggest hurdle to take is getting used to these new drugs. It often takes time to become familiar with new drugs and treatment strategies. Data from randomised clinical trials are obtained in a controlled environment with highly motivated patients, whereas ‘real world’ data are needed to confirm efficacy and safety and persuade the prescribing physician to change. Several registries now all demonstrate favourable data. The Xarelto for Prevention of Stroke in Patients with Atrial Fibrillation (XANTUS) trial, an international prospective observational study in 311 sites in Europe, Israel and Canada, included 6785 consecutive patients in whom rivaroxaban was initiated and followed the patients for almost 1 year [14]. The rate per 100 patients-years of stroke, major bleeding and mortality was 0.7, 2.1 and 1.9, respectively, which compares favourably with data of the Rivaroxaban Once Daily Oral Direct Factor Xa Inhibition Compared with Vitamin K Antagonism for Prevention of Stroke and Embolism Trial in Atrial Fibrillation (ROCKET AF) [5]. These favourable ‘real world’ data were confirmed by other registries [15, 16].

NOACs are non-inferior and in some cases superior in terms of effectiveness and safety compared with VKA in patients with non-valvular AF. Real world data are becoming more and more available and support that NOACs are safe, effective and easy to use. Despite that, the prescription rate of NOACs in the Netherlands is still low, especially compared with other countries in Europe and the rest of the world. The luxury of having well-organised specialised clinics for VKA management may be one hurdle, as well as getting used to the new treatment options. However, nowadays we see an increase in NOAC use, which persuades us that in the end NOACs will win the race.
